# Big brown bats are challenged by acoustically-guided flights through a circular tunnel of hoops

**DOI:** 10.1038/s41598-020-57632-4

**Published:** 2020-01-21

**Authors:** James A. Simmons, Patricia E. Brown, Carlos E. Vargas-Irwin, Andrea M. Simmons

**Affiliations:** 1 Department of Neuroscience, Brown University, Providence, Rhode Island 02912-9067 United States; 2 Brown-Berry Biological Consulting, Bishop, CA 93514 USA; 3 Department of Cognitive, Linguistic, and Psychological Sciences, Brown University, Providence, Rhode Island 02912-9067 United States

**Keywords:** Navigation, Animal behaviour

## Abstract

Mines and caves provide essential roosting places for bats, but often they are obstructed to prevent entry by humans. To allow bats to access their roosts, metal corrugated culvert pipes are sometimes installed. Wildlife surveys indicate, however, that bats may abandon caves having corrugated culvert entrances. Culverts may be confusing to bats due to the complex patterns of echoes returned by the regular, ring-like corrugations. We tested the hypothesis that a circular tunnel composed of successive hoops is difficult for big brown bats (*Eptesicus fuscus*) to navigate. Experiments challenged bats with flights through a tunnel of round plastic hoops or a corridor flanked left and right by rows of plastic hanging chains. The bats swerved sideways and left the pathway on more flights in the hoop tunnel compared to only rarely in the chain corridor. Even during successful flights through the hoops, bats changed the temporal patterning of their echolocation pulses to compress them into more sonar sound groups. From prior research, this active reaction is an indicator of a perceptually more difficult task. To allow bats access to mines through culverts without affecting their echolocation behavior, smoothing or masking the regular corrugations inside with concrete may be effective.

## Introduction

**Figure 1:**
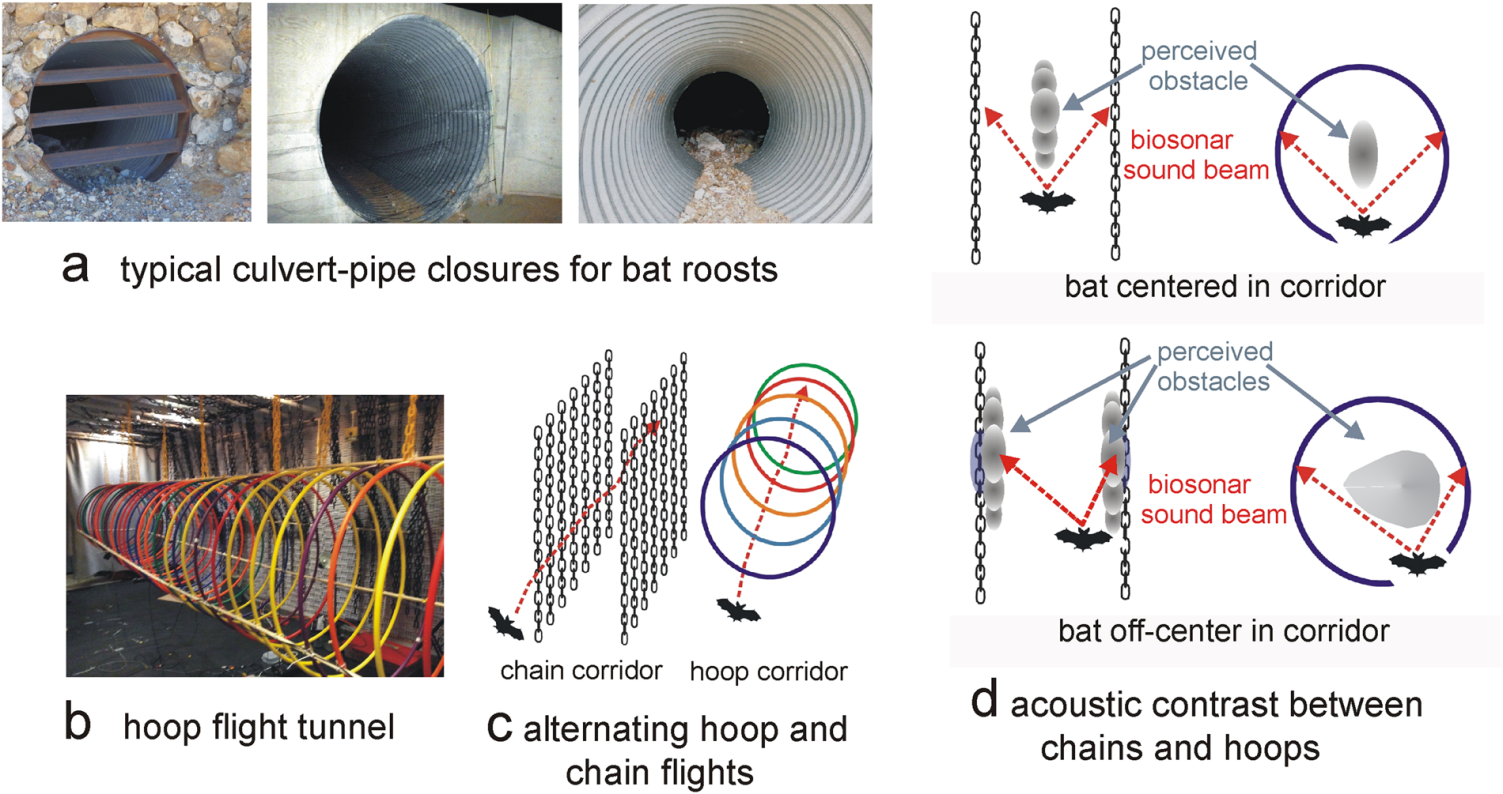
Rationale for the experiments. (**a**) Three examples of mine entrance closures using a corrugated metal pipe. Bats roosting in tunnels with corrugated culvert pipes for access may abandon the roosts, which is detrimental to bat populations in the US Southwest. (**b**) Illustration of hoop tunnel. (**c**) Design of experiments with alternating trials through the hoop tunnel (92 cm diameter) and chain corridor (70 cm corridor width). The bat’s flight path is recorded with a thermal-imaging video camera. Echolocation pulses during flight are recorded with two ultrasonic microphones placed on the end wall of the tunnel or corridor. (**d**) Explanation of acoustic rationale for special case of tunnel through hoops compared to rows of vertical chains. When the bat is positioned exactly in the center of the chain corridor (left) or hoop tunnel (right), its echolocation sounds (red arrows) impinge on the chains to left and right exactly at the same instant and return to the left and right ears simultaneously with the same spectra, leading to perception of a phantom vertically-extended obstacle (gray ellipses) located immediately to the bat’s front. Even a slight deviation of the bat’s location off-center in the chain corridor makes the echoes at the left and right ears different in arrival-times and spectra, disambiguating the phantom object so the left and right vertical chains are perceived as separate images in the chains’ true locations (gray ellipses), with no perceived obstacle to the front. In the hoops, when the bat is centered, the aggregate reflection stimulates both ears equally, so each hoop’s image is located in front of the bat (gray circle). When the bat moves off the center of the circle, the continuous reflection creates a phantom obstacle that remains in front and follows the bat’s movement.

**Figure 2:**
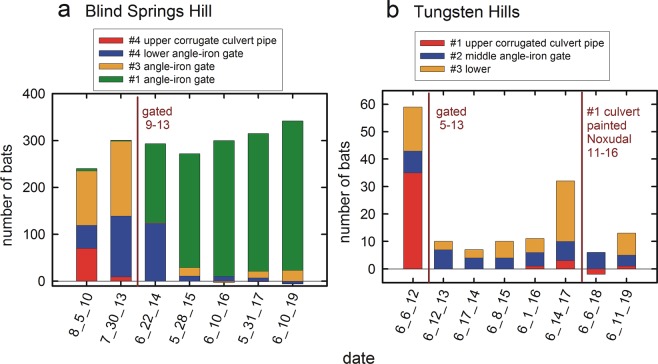
Example effects of mine blocking on bat roost activity. Counts of bats entering or exiting through several different entry points in two abandoned mines show reduced activity at entrances provided with corrugated metal culvert pipes as a component of closure to human access. (**a**) At Blind Springs Hill mine, subsequent to disturbance due to gating and permanent presence of corrugated pipe, bats ceased using the upper entrance (#4). Bat activity shifted to entrance #1, with the angle-iron gate. (**b**) At Tungsten Hills mine, gating and corrugated culvert installation of entrance #1was followed by a drop in bat activity overall and cessation of nearly all activity flying through the corrugated culvert pipe.

Natural caves and abandoned mines provide roost and hibernation sites for many North American bat species^1,2^. Because these caves and mines can pose safety risks, their entrances are often partially blocked by gates or fencing to restrict access by humans while still allowing easy access by bats. In some cases metal culvert pipes, roughly a meter in diameter and either smooth or with circular ridges (*i.e*., corrugations) to provide mechanical strength, are added to stabilize the mine entrance (Fig. 1a). Angle-iron bars sometimes are placed across the culvert pipe opening to prevent people from entering, while bats can enter by flying between the bars. Field surveys^1–4^ have assessed whether installation of gates and culverts detract bats from inhabiting mines and impact their energy budgets, mating, or flight behaviors if they do remain. Tobin *et al*.^4^ compared bat use of 28 abandoned mines in the US Southwest, some with gates and others without. Two bat species were particularly negatively affected by the presence of these gates. At several mine closures, bats have accepted smooth concrete culvert pipes with angle-iron bat gates, so neither the use of a culvert pipe as such nor the angle-iron bars necessarily deter bats from continuing to use the roost. At two of these mines (Blind Springs Hill, Mono County, CA; Tungsten Hills, Inyo County, CA), bat activity was monitored using night-vision goggles with infrared illumination and infrared-sensitive video camcorders to count bats entering or exiting the mine. At each mine, one entrance was provided with a corrugated culvert pipe while the others had either angle-iron bars or no blockage. The presence of the corrugated pipe was associated with prolonged reduced bat activity levels at those entrances (Fig. 2). Culverts may be acoustically confusing to bats due to the patterns of echoes returned by the succession of circumferential metal corrugations. The present study aims to test that hypothesis.

Echolocating bats routinely navigate through complicated scenes, including dense vegetation, the insides of roosts in caves or houses, and around buildings and other obstructions, all of which present multiple sources of overlapping echoes^5^. Obstacle-avoidance and flight performance tests in the laboratory^6–14^ have sought to mimic the spatial complexity, density, extent, and local structure of scenes that bats naturally encounter, while also offering better quantitative control of the scene’s dimensions. Using their echolocation, bats have the proven ability to negotiate even the most complex, cluttered experimental scenes^6–8,12,13^, even after exposure to high levels of background noise^14^. They do this by adapting the timing of their echolocation sounds (pulses) to the requirements of echo sequences returning from the surrounding scene. This is an effective strategy because echolocation is an active sensing system in which the timing of emitted pulses reflects the bat’s varying need to obtain updates of its surroundings while it flies through the scene. The timing of the bat’s echolocation sounds as it performs these scene-analysis tasks provides an excellent metric of a task’s cognitive difficulty as assessed by the bats themselves^6,8,9,12,13^. Higher pulse emission rates signify the need to acquire more information about the scene’s complexity to foster rapid responses to the nearest parts of the scene while also ensuring accurate orientation to the farther reaches of the scene.

In the experiments described here, we challenged big brown bats (*Eptesicus fuscus*) with flight tests through two complex scenes: a narrow corridor surrounded by vertically-hanging plastic chains that give off echoes from the bats’ left and right to mimic vegetation, and a tunnel of round plastic hula hoops designed to mimic the raised ring pattern of corrugations inside a metal culvert pipe (Fig. 1b,c). Both designs are practical for use in the laboratory; getting trees or culvert pipes into the flight test room was impractical. If the bat is stationary and positioned equidistant between the closest row of chains on its left and right, these chains return echoes that blend together into a composite echo at the bat’s ears. In this restricted situation, the resulting echoes of the chains create a phantom object located just ahead (Fig. 1d). However, a slight asymmetry to the left or right in the bat’s position results in the echoes from the nearer row of chains arriving slightly earlier than the farther row, which disambiguates the echoes into two separate sources corresponding to the actual left and right locations of the chains. Critically, the empty space to the front is now empty perceptually, and the bat can fly forward into that space. In practice, due to the bat’s motion and its inevitable slight left or right deviation from the exact center, the individual chains and the hazard-free corridor are nearly always perceptible^12–14^.

In contrast, echoes reflected by a row of open rings or hoops have unique characteristics that may interfere with the bat’s ability to perceive the empty tunnel forward, through the center of the hoops^15^. When the bat is facing the center of an annular ring or hoop, its echolocation sounds impinge all around the inside edge (Fig. 1d). This sound-reflecting edge traces a continuous circle in front of the bat, with no discrete left side, right side, top or bottom. Consequently, there are no discrete, separable reflecting segments that comprise the hoop. Instead, the bat’s broadcast registers on a continuous circle of reflecting points—a mirrorlike ring—that reflects a sound field arriving at the bat’s ears from all directions along the curve, lacking a single point-of-origin that characterizes echoes from typical shapes^15^. The *average* point of origin is in front of the bat, with no information about whether the bat should turn left or right, up or down, to avoid colliding with a real part of the ring. It creates a phantom obstacle located ahead, inside the circle, not to the sides where the actual hoop is located (Fig. 1d). When the bat deviates slightly off-center in the hoop, the phantom object just follows the bat and stays in front; it is not disambiguated because the continuous nature of the reflections follows the bat anywhere within the circle. In a tunnel made of hoops, from one hoop to the next, the bat’s exact location relative to the edges would remain difficult to perceive, with the empty space to the front always partially occupied by phantom obstacles formed from each hoop in succession. Rather than fly along the tunnel, we predicted that the bat would try to avoid the phantom obstacles, by swerving and flying out of the tunnel between two adjacent hoops. Video recordings would be expected to show the bat making abrupt turns and orienting more poorly when flying through a hoop tunnel than through a chain corridor. Moreover, the greater acoustic challenge of the hoop task ought to be manifested in faster pulse emission rates, consistent with the hypothesis that navigating through hoops is perceived as a more difficult task than navigating through chains.

## Results

**Figure 3:**
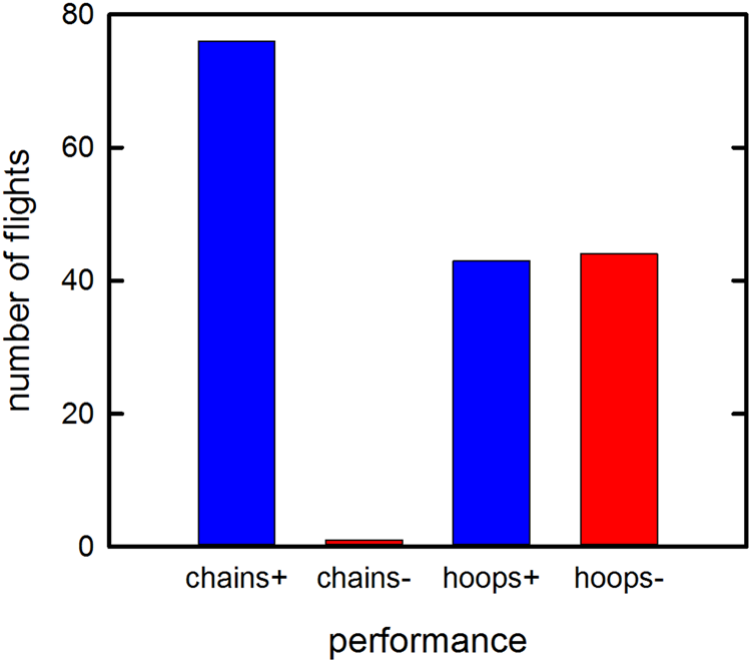
Bar graph showing the number of successful flights (+) and failed flights (−) by four bats through the chain corridor (chains) and through the hoop tunnel (hoops). Only one chain flight was unsuccessful (Table 1) because the bat landed on a chain; 51% of hoop flights were unsuccessful, either because the bat exited the hoop tunnel before reaching the far wall or collided with one of the hoops while in the tunnel.

In tests of flight guidance by echolocation, four individual bats were flown in alternate trials along the chain corridor and through the hoop tunnel (Table 1). The overall success rate for all bats was nearly perfect for the flights in the chains (76/77, or 99%) but only about half in the hoops (41/86, or 49%) (Fig. 3). This difference occurred even though the hoop tunnel was wider at its 92-cm diameter compared to the 70-cm chain corridor width. Differences in performance between the chain flights and the hoop flights are statistically significant (Friedman repeated measures analysis of variance on ranks: χ^2^ = 16.64, *P* < 0.001). Paired comparisons using the Student-Newman-Keuls method showed differences in ranks (*P* < 0.05) for all comparisons except hoop successes and failures, which were statistically equal.

**Table 1:** Performance of the four bats in flights along a corridor between rows of chains and along a tunnel through hoops, on each of the two flight days (Day 1/Day 2).

	Chains success (Day 1/Day 2)	Chains fail (Day 1/Day 2)	Hoops success (Day 1/Day 2)	Hoops fail (Day 1/Day 2)
Bat A	8/10	1/0	7/10	3/0
Bat B	10/10	0/0	0/3	12/7
Bat C	10/10	0/0	7/8	3/2
Bat D	8/10	0/0	2/6	10/7

Success = number of successful flights with landing on far wall. Fail = number of failed flights with early exit, or colliding with any hoops or chains within the tunnel or corridor. All bats had more successes on chain flights compared to hoop flights on both days.

All bats showed excellent performance (success or failure) in the chain corridor, with only one bat committing one error on the first day of testing (Table 1). On the other hand, there were more failed flights as well as considerable individual differences in performance in the hoops. Averaged over both testing days, two of the bats showed fewer errors (Bats A and C, 15% and 25% errors, respectively) than the other two (Bats D and B, 75% and 86% errors, respectively). Even in the cases of Bats A and C, the percent of errors on the hoop flights (15% and 25%) exceed those on the chain flights (0.05% and 0%, respectively). Still, all bats improved their performance in the hoop tunnel from the first to the second day of testing, with one bat (Bat A) reaching 100% correct performance.

**Figure 4:**
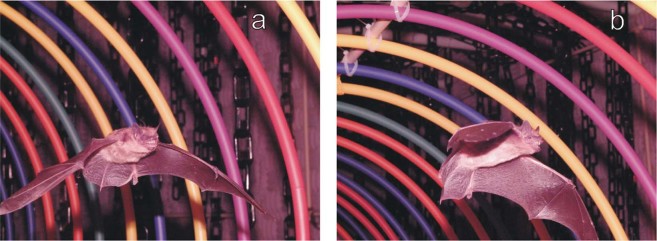
Photographs of big brown bat flying in the hoop tunnel. (**a**) Bat proceeding along the tunnel towards the far wall. (**b**) Bat swerving to one side before exiting the tunnel by flying between adjacent hoops. The vertical chains visible in background demarcate the chain corridor used for alternate trials (see Fig. 1c).

As observed on thermal-imaging video, successful flights in both the chain corridor and the hoop tunnel (Supplementary Video 1) involved the bat flying through to the far wall (Fig. 4a). The bats in failed flights in the hoop tunnel swerved sideways to exit the tunnel before reaching the end (Fig. 4b; Supplementary Video 2). These often involved collisions with one of the hoops as the bat tried to get out between two of them. In the single failed flight through the chains, the bat hit a chain while attempting to land on it. Nevertheless, in spite of the prevalence of multiple failures in the hoop tunnel, the bats began each of the alternating hoop and chain flights with no sign of reluctance, initiating flight in both experimental conditions. Surprisingly, individual chain flights that followed each failed hoop flight were themselves successful, indicating no lingering deterrent effect of the failures.

**Figure 5:**
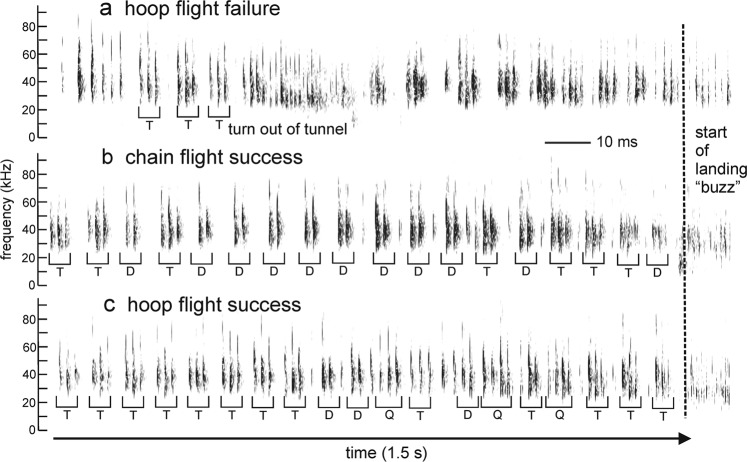
Spectrograms of pulse emissions by Bat D during three successive flights. All three examples are 1.5 s in duration and end with landing. (**a**) A flight through the hoop tunnel with early exit, with the bat hitting a hoop before flying along the adjacent chain corridor to land on the far wall (see Fig. 1c). IPIs consist of individual pulses, followed by triplets (T, short interval between three adjacent pulses, longer interval between other groups). A rapid burst of sounds (a landing buzz) is seen as the bat exits the tunnel and lands on the wall. IPIs are predominately in triplets when finishing the flight through the chains. (**b**) A successful flight through the chain corridor to the far wall, ending with a buzz as the bat lands. IPIs consist of many sound group doublets (D; short interval between two adjacent pulses, longer interval between other pairs). (**c**) A successful flight through the hoop tunnel to the far wall. IPIs consist of triplet sound groups near the beginning of the flight, a few doublets, and quadruplet sound groups (Q) near the end.

**Figure 6:**
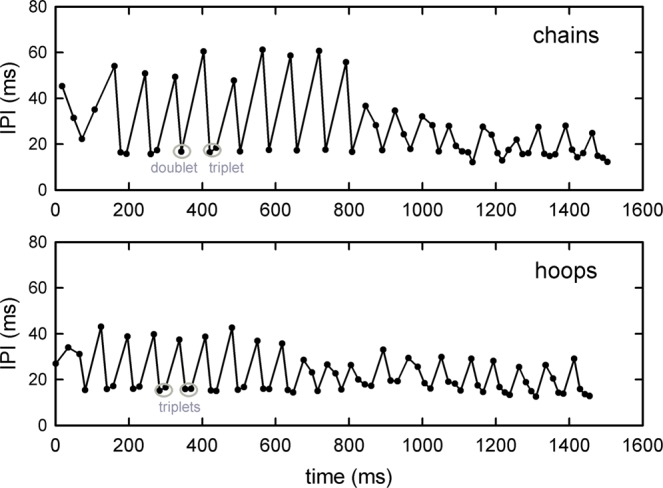
Progression of IPIs for the two sound sequences in Fig. 5b (successful chain flight) and 5c (successful hoop flight). Alternating single long and single short intervals signify sound-group doublets and alternating single long and double short intervals signify triplets.

The high proportion of failed flights suggests that the hoop tunnel was more difficult for the bats to negotiate. This possibility is supported by examining a different criterion—the bat’s pattern of pulse emissions during the flights (Figs. 5, 6). In one example of a failed hoop flight (Fig. 5a), the bat exited out of the tunnel about one-third of the way to the far wall by flying between two adjacent hoops while hitting one, flew into the chain corridor, and then completed its flight in that chain corridor. Note the buzz of closely-spaced pulses when the bat passed between the hoops on its way out of the tunnel. In a successful flight through the chains (Fig. 5b) and in a successful flight through the hoops (Fig. 5c), the same bat flew without incident and landed on the far wall, concluding the flight with a landing buzz of rapidly-emitted pulses. The sequences of pulses in these different conditions differ, however. In both sound sequences, IPIs alternate between long and short intervals, but either with only one long and short IPI, which indicates a doublet sonar sound group (D), or with one long and two short IPIs, which indicates a triplet sonar sound group (T) [as defined consistent with the criteria in (9)]. In the successful chain flight, more pulses are grouped in pairs (doublets, D; Figs. 5b, 6), while in the successful hoop flight, more pulses are grouped in triplets (T) (Figs. 5c, 6). Doublets predominate in the successful chain flight, while triplets predominate in the successful hoop flight. In the latter part of the hoop flight, several quadruplet sound groups (Q) are also present. For both flights, the short IPIs are in the same range of 15–20 ms. But longer IPIs differ in these two flights, ranging between 35–40 ms in the hoop flight and 50–60 ms in the chain flight.

**Figure 7:**
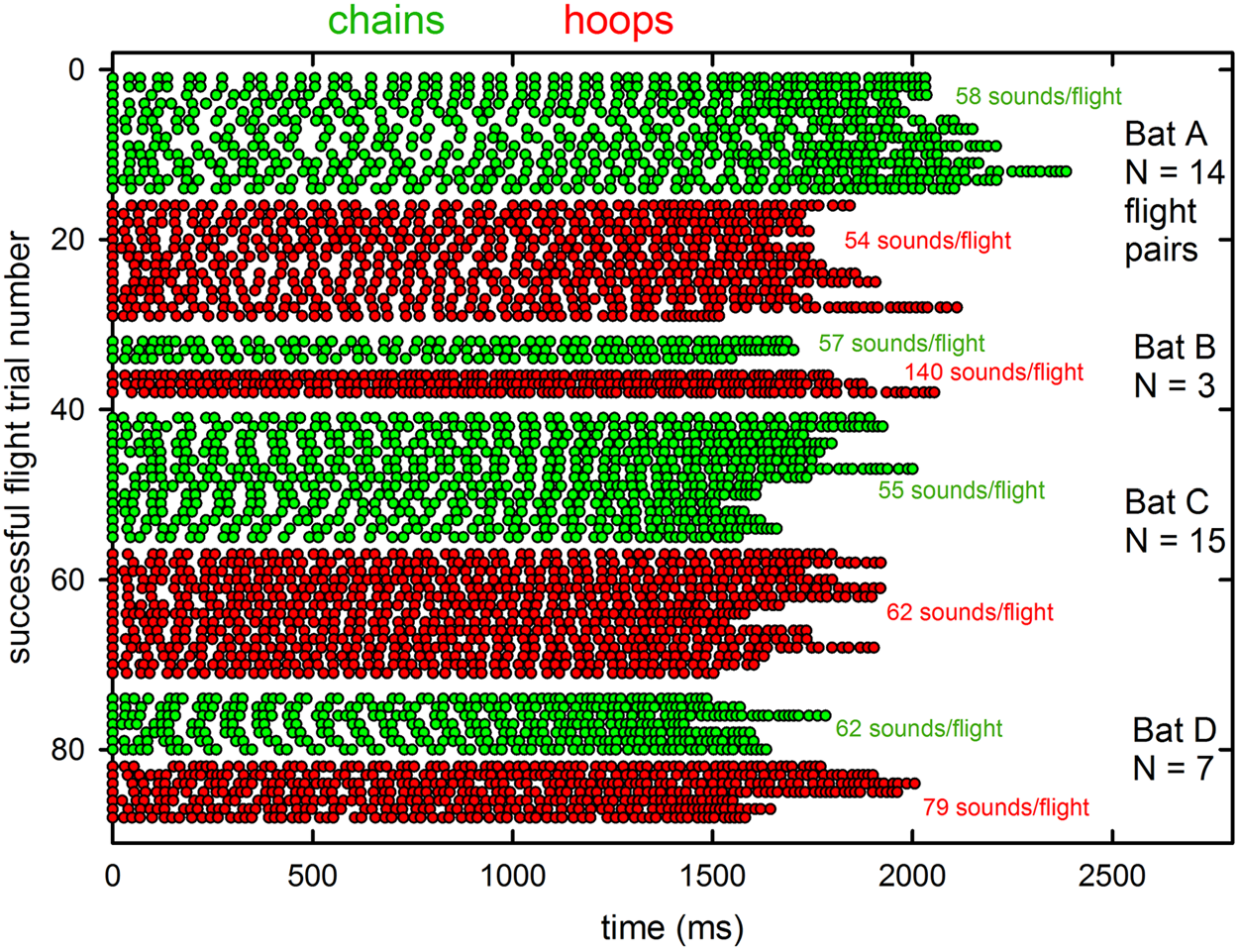
Pulse emission patterns (dots show timing of individual emissions in each flight—horizontal row) for successful flight trials through the chain corridor (green) or through the hoop tunnel (red). In these analyses, the numbers of successful chain and hoop flights were matched for each bat. Because failed flights were more common in the hoop tunnel, for each successful hoop flight, the next chain flight was paired with that hoop flight for analysis. The proximity of the hoop and chain flights in each day’s testing was intended to minimize comparing trials that took place earlier in the session with trials that occurred later.

For quantitative comparison of pulse emissions during chain and hoop flights, we balanced the numbers of flights by choosing, for each bat, the next successful chain flight after each successful hoop flight. This resulted in a sample of 39 chain flights and 39 hoop flights across all four bats. Repeated measures analysis of variance showed that significantly larger numbers of pulses are emitted in successful hoop (mean 83.6) than in successful chain (mean 57.9) flights [F_1, 35_ = 319.8, *P* < 0.0001]. This pattern of greater numbers of pulses in hoop flights was consistent in the data from three of the four bats, with Bat A showing a small reverse effect (Fig. 7).

**Figure 8:**
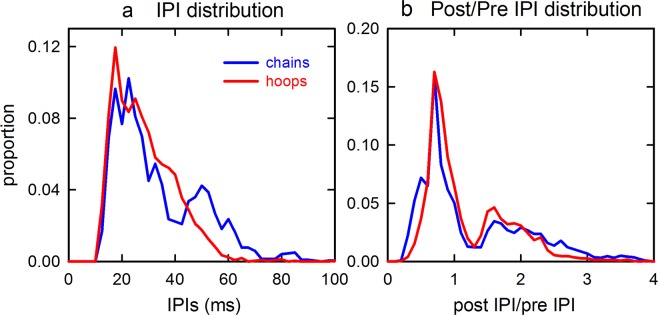
(**a**) Histograms showing the distribution of IPIs for the chain and hoop flights from Fig. 7. Mean IPIs in hoop flights are significantly shorter than those in chain flights. For chain flights, the bimodal distribution (with peaks at 20–25 ms and 50 ms) reflects alternating short and long IPIs, characteristic of doublet sonar sound groups. For hoop flights, the shift from a clear second peak at 50 ms to a single broader peak at 20–30 ms reflects multiple shorter IPIs for triplet and quadruplet sonar sound groups. (**b**) Histograms for the distributions of the ratio between the IPI after each pulse (post-IPI) and the IPI before each pulse (pre-IPI) in both the chain flights and hoop flights.

Over all bats, the IPIs for the pulse sequences have different distributions in chain and hoop flights. First, the timing of individual pulses in the sound sequences for these flights for each individual bat (Fig. 7) shows greater regularity in timing of pulse emissions across chain flights compared to hoop flights. Pulse times are similar across several adjacent chain flights, leading to a pattern of data points aligning vertically in the plot. The timing of pulses in the hoop flights appears less organized and more irregular, with fewer data points aligning vertically. The significantly greater number of pulses emitted during hoop flights makes the pulse patterns denser, as well, which shortens the mean IPIs. Repeated measures analysis of variance show that IPIs are significantly shorter in hoop flights (mean IPI = 27.2, N = 2613) than in chain flights (mean IPI = 32.4, N = 2201) [F_1,2140_ = 108.9, *P* < 0.0001]. This pattern of shorter IPIs in hoop flights was apparent in the data from all four bats. The underlying distributions of IPIs for both the chain and the hoop conditions are both skewed to low IPIs, with a long tail extending to 70–90 ms. But these distributions of IPIs do not overlap (Fig. 8a). Differences in the shapes of the IPI distributions are statistically significant (two-sample Kolmogorov-Smirnov test: Z = 6.132, *P* < 0.0001).

By themselves, the IPI distributions conceal a prominent internal organization in the form of regular zig-zag alternation between one long and one short interval (for pairs of pulses in doublets), or from one long and two short intervals (for three pulses in triplets) (Fig. 6). As previously described^12,13^, the *ratio* between each measured IPI from one pulse to the next (post-IPI) and the previous IPI in the sequence (pre-IPI) relative to any given pulse (post-IPI/pre-IPI) registers the underlying constancy of the alternation pattern. The distributions of post- to pre-IPI ratios for the chain and hoop fights from all four bats are strongly bimodal (Fig. 8b), which reflects the short-to-long IPI alternations that dominate the sound sequences. (If the IPIs were, on average, the same from one pulse to the next, the ratios should be distributed around 1.0; instead, they are distributed at 0.7 and 1.6.) These distributions do not differ significantly (two-sample Kolmogorov-Smirnov test: Z = 0.98; *P* = 0.29), confirming the ubiquity of IPI alternations.

**Figure 9:**
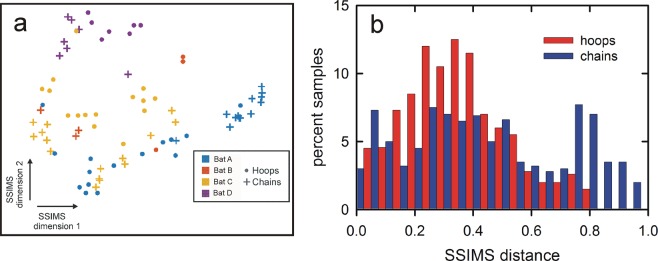
(**a)** Projection of the multidimensional distribution of SSIMS data points onto a two-dimensional plot showing the two most prominent dimensions (dimension 1, x axis; dimension 2, y axis) of the SSIMS differences between all pairs of hoop and chain flight IPIs. **(b)** Histograms for the SSIMS distances on dimensions 1 and 2 between all pairs of flights in the hoop tunnel (red) and the chain corridor (blue) for four bats. SSIMS distances in the hoop IPI distribution are significantly shorter than in the chain IPI distribution.

To further compare the hoop and chain IPI sequences strictly as a time series of events, the Spike Train Similarity Space analysis (SSIMS)^13,16^ was applied to the paired sequential hoop and chain IPI sequences displayed in Fig. 7. The SSIMS method derives a “cost” measure required to convert one sequence in each pair to the other. The result is a numerical estimate for the amount of “work” required to add or delete individual pulses, or to shift some pulses to different times, so that one sequence in a pair becomes identical to the other^17^. These pairwise SSIMS costs for hoop and chain flights form a multidimensional distribution of the SSIMS distances between both the four individual bats (Bats A-D) and the two flight tasks (hoops *vs* chains). These distances then are projected onto a plane defined by the two most prominent multidimensional features as horizontal axis (SSIMS dimension 1) and vertical axis (SSIMS dimension 2) as determined using the t-SNE dimensionality reduction algorithm^18^. The data points representing the individual pulse sequences emitted by each of the four bats are widely separated for both the hoop and the chain conditions (Fig. 9a), reflecting the prominent individual differences in performance (success or failure) that are evident in Table 1. Nevertheless, summarized over all bats, the data points are more closely spaced together (smaller SSIMS differences) for the hoop flights than the chain flights. The distribution of distances in SSIMS space between pulse sequences in hoops is tighter than the spread for chains, so that the peak of the hoop distribution is shifted towards smaller SSIMS distances (Fig. 9b). The mean SSIMS bat-to-bat distance for the hoop flights is 0.34 and the mean for the chain flights is 0.49. This difference is significantly significant (paired t-test; t = 3.19, df = 206, *P* = 0.0016). The two distributions of SSIMS distances differ significantly (Wilcoxon signed-rank test; W = 4274, *P* < 0.009). Thus, the compression of IPI values to shorter SSIMS distances for the hoop flights is reflected both in terms of the mean and in terms of the distribution’s shape.

## Discussion

The origin of these experiments was twofold; one, the observations that some species of bats abandon roosts that are outfitted with metal corrugated culvert pipes^1,4^; Fig. 2, and two, the suspicion that the annular ribs in the inside of these corrugated culverts provide spatially diffuse, ambiguous reflections with regard to the localizability of the echo sources, that might disrupt echolocation-guided flight by introducing acoustic confusion. The hoop flights were more difficult for big brown bats to complete successfully than were the chain flights. The difficulty of the hoop task may be a consequence of acoustic geometry of the ring shape itself^15^ because it is not the size of the space that the bat had to transit.

When echolocating big brown bats encounter greater task difficulty, they emit broadcasts with more sonar sound groups, shorter mean IPIs, and specific changes in IPI distributions^6–14^. This effect was confirmed in the present data, when comparing broadcasts in chain and in hoop flights. These differences in pulse sequences were seen in all bats, even though some bats were more successful than others in navigating through the hoops. Individual differences are common in laboratory flight experiments with big brown bats^12–14^. Interestingly, even though the mean IPIs themselves are shorter for the hoops than for the chains and have a more concentrated distribution, their corresponding post- to pre-IPI ratios are similar. This result suggests that a similar overarching pulse-timing rule or strategy is being used for both chain and hoop flights. In addition, individual differences in pulse sequences, as shown by the SSIMS analysis, decreased in the hoop flights compared to the chain flights. This suggests that task difficulty moves the bats within a space defined by the strategy instead of changing to a different strategy. The greater concentration of SSIMS distances in hoops compared to chains supports this suggestion.

It is important to note that the four bats in these experiments had prior experience flying in chain corridors but not in hoop tunnels^12,13^. This raises the question as to whether this previous experience led to better performance in the chain corridor, aside from the different patterns of echoes produced by the chains and by the hoops. Wheeler *et al*.^12^ reported that the same four bats in their first experience in chain corridors had a mean success rate of 97.6% (range 96.8–98.1%) navigating through dense corridors with widths of 40, 70, and 100 cm. No practice effects were reported in those experiments; that is, bats were as successful on the first day of flights as on the last. Practice effects cannot be ruled out in the present experiment, because bats were tested only for two days and then in an alternating sequence of chain and hoop flights. All bats showed improved performance on the second day in the hoop tunnel, but it is not known if this improvement is sustainable, or if all bats would reach the same level of performance in the hoops as in the chains. The observation that two bats showed good performance in the hoops indicates that the presence of hoops, while challenging, is not a universal acoustic deterrent. Of course, plastic hula hoops are not corrugated metal rings, so future work should explore other circular configurations, with and without corrugations, to further explore the limits of bats’ performance.

As shown in other experiments—especially tests of performance in what might be considered difficult conditions (obstacle avoidance, flight in complex surroundings, resistance to interference, resistance to temporary hearing loss due to noise exposure, discrimination of small differences in echoes or targets)^5,19^—echolocation is an extremely sensitive and versatile sensing system. Still, in the natural environment, the presence of acoustic confusion at entrances to caves or mines might deter bats from attempting to inhabit those mines. The finding that flights through the hoop tunnel are more challenging than flights in even narrower chain corridors indicates that ring-like configuration of acoustic reflections likely pose a difficult problem for the bat to surmount. Resource managers might consider breaking up the reflective structure of corrugated culvert pipes by spraying on concrete inside the pipe to help mitigate the deleterious acoustic effect of the ribs, so that bats might enter and exit these mines more easily.

## Materials and Methods

### Animals

Four wild-caught, adult big brown bats (labeled here as Bats A, B, C, and D), in captivity for two years, participated in these experiments. They were socially-housed in a temperature- and humidity-controlled colony room (22–24 °C, 40–60% relative humidity) on a reverse 12:12 circadian cycle. In their home cages, bats had unlimited access to vitamin-enriched water. During flights, they were offered pieces of live mealworms, up to their daily food allotment, as rewards for correct performance; if necessary, extra food was offered after completion of flights to maintain bats’ body weights within the range of 15–18 g. Bats were individually recognized by a distinct pattern of haircuts on their backs. Numbers of bats permitted for collection are strictly limited and monitored by the State of Rhode Island Department of Environmental Management, thus restricting the numbers available for capture and experimentation. All methods were carried out in accordance with relevant federal guidelines and regulations. All experimental protocols were approved by the Brown University Institutional Animal Care and Use Committee.

### Flight room configuration

Experiments were conducted in a custom-built flight room (8.3 m × 4.3 m × 2.7 m) whose walls and ceiling were lined with sound-absorbent foam (SONEX) to attenuate echoes. The room was illuminated by infrared LEDs mounted on the walls except for one dim light (90 lx) positioned on the ceiling to aid the experimenter in releasing the bat for flights. Flights were conducted within either a hoop tunnel (Fig. 1b) or a chain corridor, both of which were 5.4 m long and adjacent to each other (Fig. 1c). The hoop tunnel was a 92 cm diameter opening surrounded by a row of 36 plastic hula hoops, each 90 cm in diameter, spaced at regular intervals of 15 cm. The chain corridor was a 70 cm diameter opening surrounded by rows of black plastic chains (link size 4.0 cm wide, 7.5 cm long, 1.0 cm thick) spaced at regular intervals of 15 cm.

The bat’s flight path through the hoops or the chains was recorded with a thermal-imaging video camera (Photon 320) located 60 cm in front of the hoop tunnel. Sounds emitted by the bats during flights were recorded with two ultrasonic microphones (Knowles SMG-0291) placed on the far wall, one directly opposite the exit of the hoop tunnel and the other directly opposite the exit of the chain corridor. Both microphones were mounted on custom-built preamplifier and high-pass filter boards fitted with a 20-cm square foam baffle to minimize backscatter. The output of each microphone was recorded on one channel of a TEAC digital recorder (Model HD-P2) at a sampling rate of 192 kHz and saved as stereo.wav files.

### Experimental procedure

All four bats participated previously in experiments in which they were challenged successfully with flights through chain corridors of different widths, configurations, and curvatures^12,13^. None of the bats had any previous experience with the hoop tunnel.

Experiments took place during the bats’ subjective nights, within 3–4 hours of lights off. Each bat was tested on two separate flight days. On each day, one experimenter released the bat by hand 60 cm in front of the entrance of either the hoop tunnel or the chain corridor. Microphone recordings were manually started by a second experimenter at the time of the bat’s release and then manually stopped when the bat either landed on the far wall or otherwise ended its flight. A successful flight was defined as flying through the tunnel or the corridor to the far wall without exiting or without hitting any hoops or chains along the way. A failed flight was defined as striking or landing on a hoop or a chain, or exiting the tunnel or corridor before completing the flight.

At the beginning of each experiment, each bat was allowed three practice flights through the chain corridor. This was to ensure that the bat was awake and motivated to perform in a task that should elicit good performance. Then, the bat was flown alternately through the chains and through the hoops, for a total of 19–22 total flights per day, with the goal of achieving equal numbers of hoop and chain flights (Table 1). In practice, however, because performance in the hoop tunnel was more variable, bats were given a few extra hoop flights to assess whether poor performance was due to the bat itself or to experimenter error in releasing the animal. Numbers of flights also varied according to the number of food rewards available as a result of the bat’s food allotment and performance, and on its continued motivation to fly.

### Data analysis

All statistical tests were performed in SPSS v. 25 (IBM SPSS Statistics). Bats’ performance was quantified as numbers of successful and failed flights, with statistical significance assessed using Friedman repeated measures analysis of variance by ranks. To assess any changes in sound emission patterns and thus the potential perceptual difficulty of the flight task to the bat, the timing of individual pulses was extracted from the entire sound sequence during each flight. The.wav files from the TEAC recorder were high-pass filtered at 15 kHz to reduce ambient noise. The files for each individual successful flight were manually trimmed to a standard time interval of 1.5 s, beginning with the onset of the unambiguous “landing buzz” of low amplitude, high repetition rate pulses marking the bat’s arrival at the far wall of the room (Fig. 5), and then moving backwards in time. Trimming included only those sounds the bat emitted during sustained flight (*i.e*., excluding those emitted before release or after landing). This procedure also eliminated any variability in the length of the recording sequence produced by the manual onset/offset of the TEAC recorder. Failed flights were shorter than successful flights, because in those cases the bat did not reach the far wall. Recordings of these flights were truncated manually backwards from the time at which the bat made the error, as indicated by a landing-type buzz when the bat negotiated the gap between adjacent hoops. The estimated time at which the bat entered the tunnel or the corridor is indicated by very long IPIs (>80 ms) and the absence of sonar sound groups^6,14^. The individual who trimmed the audio files worked on coded files and was unaware of the hypothesis of this study. For each trimmed segment of sounds, a custom-written MATLAB (2014a) script determined the time of occurrence of each individual pulse, the total numbers of pulses, their IPIs, and the ratio of pre- to post-IPIs. Differences in pulse numbers and in IPIs between hoop and chain flights were analyzed by repeated measures analysis of variance.

To compare the distribution of IPIs between hoop and chain flights, we paired each successful hoop flight with the subsequent successful chain flight. These IPI distributions were plotted as the relationship between post-IPI and pre-IPI intervals (that is, the interval between each subsequent pulse compared to each preceding pulse). Differences between hoop and chain flights in IPI and IPI ratio distributions were analyzed using two-sample Kolmogorov-Smirnov tests. IPI distributions in successful flights were also analyzed using the SSIMS method, a model-free approach to comparing time-series records, as previously described^12,13^. SSIMS was originally developed for determining the similarity or dissimilarity of neural spike time sequences^16^. Each IPI sequence consists of a record of the time-of-occurrence of the individual pulses during one successful flight. The SSIMS method computes pairwise comparisons of all the hoop and chain flights to determine for each pairing how much “work” is needed to convert one member of each pair into the other. That is, for each pair of flights, the method assesses a cost for adding or deleting individual time values to a sequence to account for there being more sounds or fewer sounds in one sequence compared to the other. Another cost is assessed for moving individual sounds present in both records so they coincide in time. The collective cost represents the degree of dissimilarity between the two records. This dissimilarity is then expressed as distance between all pairs of flights in a multidimensional space.

## Supplementary information

Supplementary video 1.

Supplementary video 2.

## Data Availability

Data are deposited in the Brown University data archive 10.26300/81t0-6c47.
